# Effect of the early-life nutritional environment on fecundity and fertility of mammals

**DOI:** 10.1098/rstb.2009.0121

**Published:** 2009-11-27

**Authors:** D. S. Gardner, S. E. Ozanne, K. D. Sinclair

**Affiliations:** 1 School of Veterinary Medicine and Science, University of Nottingham, Loughborough LE12 5RA, UK; 2 School of Biosciences, Sutton Bonington Campus, University of Nottingham, Loughborough LE12 5RA, UK; 3 Institute of Metabolic Science, University of Cambridge, Addenbrooke's Hospital, Box 289, Level 4, Cambridge CB2 0QQ, UK

**Keywords:** nutrition, programming, reproduction, fertility

## Abstract

The early-life developmental environment is instrumental in shaping our overall adult health and well-being. Early-life diet and endocrine exposure may independently, or in concert with our genetic constitution, induce a pathophysiological process that amplifies with age and leads to premature morbidity and mortality. Recently, this has become known as ‘programming’ but is akin to ‘maternal effects’ described for many years in the biological sciences and is defined as any influence that acts during critical developmental windows to induce long-term changes in the organisms' phenotype. To date, such delayed maternal effects have largely been characterized in terms of susceptibility to cardiovascular or metabolic disease. Here, we review evidence from experimental animal species, non-human primates and man for an effect of the early-life nutritional environment on adult fecundity and fertility. In addition, using a database of pedigree sheep, we also specifically test the hypothesis that being born small for gestational age with or without post-natal growth acceleration directly programmes fertility. We conclude that there is a lack of compelling evidence to suggest pre-natal undernutrition may directly reduce adult fecundity and fertility, but may exert some effects secondarily via an increased incidence of ‘metabolic syndrome’. Possible effects of being born relatively large on subsequent fecundity and fertility warrant further investigation.

## 1. Introduction

The current conceptual framework, that the phenotype of an adult individual can be influenced by events that occur earlier in life, e.g. either *in utero* and/or during infancy, can trace its origins to the pioneering studies of investigators such as John Hammond and his associates at Cambridge (Hammond 1932; Hammond *et al*. 1976), and Robert McCance and Elsie Widdowson (Widdowson 1970; McCance & Widdowson 1974). Although one can always find earlier studies that recognized the importance of ‘maternal effects’ in biology (Forsdahl 1977), the clinical significance of variations in the *in utero* growth process in humans was only fully realized following the publication of epidemiological data collated by David Barker and Clive Osmond at the MRC Environmental Epidemiological Unit, Southampton University (Barker & Osmond 1986, 1987; Barker *et al*. 1989). Using birth weight as a proxy for *in utero* development in an epidemiological study, these authors established a link to death by ischaemic heart disease in the aged population. Further studies were to establish inverse relationships between birth weight and the incidence of stroke, Type 2 diabetes and dyslipidaemia, and these phenomena, often collectively referred to as metabolic syndrome, led to the ‘foetal origins’ (Barker 1995) or later to be called ‘developmental origins of adult health and disease’ or ‘DOHaD’ hypothesis (Gluckman & Hanson 2004; McMillen & Robinson 2005). The central tenet of this hypothesis, that low birth weight is associated with late-onset disease, however, has since been challenged on the basis that early statistical models tended to adjust for the current weight of the cohort of individuals under investigation (Lucas *et al*. 1999; Huxley *et al*. 2002). Such analyses often confound the effects of *in utero* development with early infant growth, and so were inclined to exaggerate the importance of the former. Rather, it is axiomatic that birth is a major event for both mother and baby but, in essence, should be viewed statistically as simply a phase that transitions from pre-natal to post-natal growth. When viewed this way, it follows that the rate of early infant growth, often referred to as ‘centile crossing’, is at least as important as the rate of *in utero* growth; indeed, it is inextricably linked (Eriksson *et al*. 2000; Barker *et al*. 2005; Ben Shlomo *et al*. 2008). Nevertheless, the general consensus based on an overwhelming body of epidemiological evidence among different human populations (Kaijser *et al*. 2008; Whincup *et al*. 2008) and from direct-interventional studies with animals (Langley-Evans 2004) supports the general concept that malnourishment restricted either to *in utero* development or during infancy can predispose to late-onset non-communicable disease in adult individuals.

The major non-communicable human diseases of our time are cardiovascular and metabolic, each of which is susceptible to ‘developmental programming’ (Armitage *et al*. 2004; Ojeda *et al*. 2008). By contrast, relatively little is known about the effects of variations in maternal diet during pregnancy and lactation on subsequent fertility of offspring (Gardner *et al*. 2008). This is in stark contrast to the effects of exposure either to environmentally prevalent endocrine-disrupting chemicals (Toppari *et al*. 1996; Henley & Korach 2006) or to excessive reproductive hormones during critical ‘sensitive’ periods of development of the male or female reproductive axes (Manikkam *et al*. 2008; Veiga-Lopez *et al*. 2008). The effects of these agents have been extensively reviewed elsewhere, and so are beyond the scope of the current article. Instead, we focus on so-called maternal effects: that is, how variations in maternal environment, usually represented as variations in nutrient intake, may impact upon subsequent reproductive function of the adult offspring; defined as effects on *fertility* (i.e. a productive term representing the actual numbers of offspring produced) or *fecundity* (i.e. the biological capacity for reproduction). We critically review the existing literature, but begin by presenting some new data for a possible effect of early environment (using birth weight as a proxy measure) adjusted for post-natal environment (i.e. growth rate) and controlling for a possible contribution from the shared genomes of mother and offspring. The new data come from five flocks of pedigree Suffolk sheep (*n* = 2427 ewes) and were analysed using generalized linear mixed model procedures, therefore allowing determination of fixed effects (e.g. fertility) but controlling (blocking) for random effects such as different flocks and shared parentage (i.e. ewe and ram effects). The dataset was kindly provided by Signet Breeding Services (Meat and Livestock Commission, Milton Keynes, UK).

## 2. Fertility in pedigree suffolk ewes

### (a) Effects of birth weight

**Figure 1.**
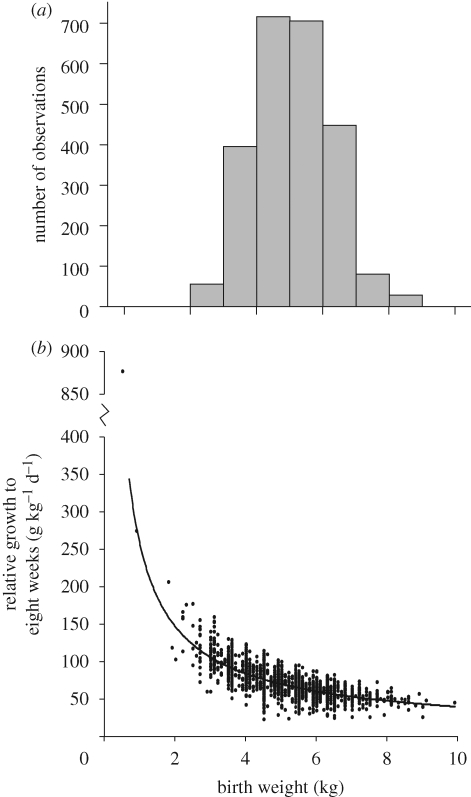
(*a*) Birth weight of pedigree Suffolk ewes born as singletons, and (*b*) their relative growth to eight weeks of age. (*a*) Data extend to 2427 observations of birth weight or (*b*) birth weight adjusted (relative growth) to eight weeks of age (g kg^−1^ d^−1^). The ewes as lambs were weaned at eight weeks of age.

**Figure 2.**
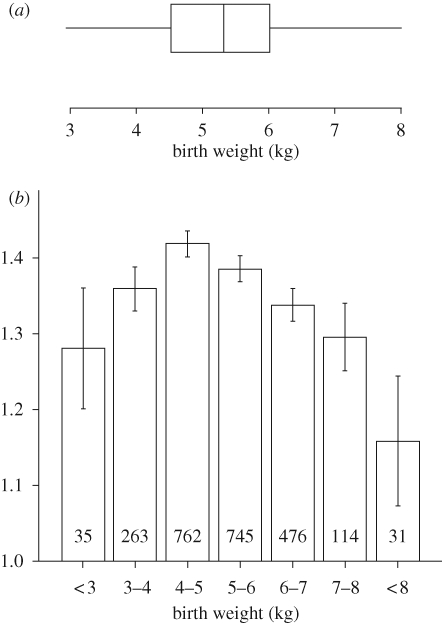
(*a*) Box and whisker plot of birth weight of ewes born singletons and (*b*) their lifetime fecundity expressed as the average number of lambs per litter. Boxplot data are medians with interquartile ranges. Litter size expressed as means ± s.e.m. Values in boxes represent the number of ewes in that category. Quadratic relationship between birth weight and litter size was significant (*p* < 0.001). Adapted from Sims *et al*. (2008).

These ewes were all born as singletons and the population exhibited a fourfold natural variation in birth weight (figure 1*a*). Lambs that were born relatively small for gestational age (SGA) (less than 3 kg), defined as being 2 s.d. below the mean birth weight (5.15 ± 1.1 kg), only exhibited early catch-up growth to weaning at eight weeks of age (figure 1*b*) and not thereafter (i.e. from eight to 20 weeks of age; data not shown). As adult ewes, the sheep went on to have between one and 11 litters (median, 3 (1–4) lower–upper quartiles) and from 1–19 lambs (median, 3 (2–5) lower–upper quartiles). When the ewes were grouped by their birth weight into standard 1 kg categories, and these groups were plotted against their lifetime fertility (derived as average number of lambs per litter), then an interesting and significant (*F* = 12.1, *p* < 0.001) quadratic relationship emerged (figure 2). It would appear that being born relatively small or relatively large (i.e. +2 s.d. = 7.4 kg) reduces fertility in female sheep, the effect being most pronounced as the lambs get progressively larger at birth. Importantly, and to reiterate, this effect was quadratic and so could explain why some studies that simply assessed the effects of low versus high nutrient intakes during pregnancy or low versus high birth weights failed to detect an effect on fecundity (Gunn *et al*. 1995; Da Silva *et al*. 2001, 2003) as discussed later. To emphasize this point, note the similarity in fertility (approx. 1.29 lambs per litter) between females born at less than 3 kg or greater than 7 kg; each of which falls ±2 s.d. from mean birth weight in these Suffolk sheep.

### (b) Effects of early post-natal growth

**Figure 3.**
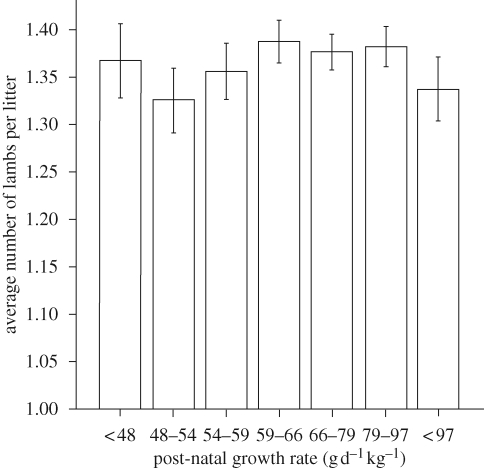
The effect of relative post-natal growth rate (up to eight weeks) on lifetime fecundity of female pedigree Suffolk sheep. Data are predicted means ± s.e.m. for singleton female lambs. Relative growth rate is calculated as g d^−1^ kg^−1^ birth weight.

From previous studies, it appears that post-natal growth may independently induce a developmentally programmed phenotype (reviewed by Gardner *et al*. 2008). We therefore assessed the extent to which this might apply to our measure of fecundity in the current cohort of pedigree Suffolk sheep. As illustrated in figure 1*b*, ewe lambs growth restricted at birth had a tendency to regress back to the mean growth rate for that breed during the first eight weeks of life. Since absolute rates of growth will increase linearly with birth weight as a simple mathematical function, we expressed early post-natal growth rate relative to birth weight (as g d^−1^ kg^−1^ birth weight) and similarly plotted this value against our value for fertility (i.e. lifetime average number of lambs per litter) in the sheep. Clearly, for this cohort of singleton females, post-natal growth rate did not influence future reproductive success (figure 3). Taken together, it would appear that the pre-natal environment leading to a female lamb that is born either relatively small or large has a much greater influence on fertility in sheep than post-natal growth during infancy to weaning.

**Figure 4.**
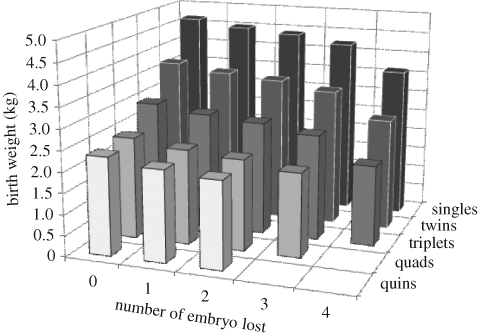
The effect of early embryo loss on birth weight of lambs born as singletons. Relative to singles, birth weight reduces with increasing litter size. However, within each litter size category, birth weight also reduces as early embryo loss increases. As an example, for singletons, birth weight reduces by on average 1.05 kg if that singleton was of a litter that experienced a loss of four embryos around the time of implantation. Adapted from Hinch *et al*. (1985).

However, an important assumption in this dataset, and indeed in any dataset that reports on singleton births, is that each singleton at birth arose following the implantation of a single oocyte. For sheep, at least, we know that this is not always the case. Hinch *et al*. (1985) estimated that the birth weight of 8 per cent of lambs born as singletons was depressed by as much as 300 g as a result of intrauterine sibling death and early embryo loss (figure 4). Therefore, there is potential for a residual genetic effect that may explain why some lambs born relatively larger at birth may have more of a negative effect on fertility, as the smaller lambs may actually have come from a multiple pregnancy, and therefore have inherited and express genes for increased litter size. To account for this in the current cohort, we removed the lightest 8 per cent of lambs and reanalysed the data; we saw no change in the significance of the quadratic relationship depicted in figure 1*b*. It is unlikely therefore that the curvilinear relationship observed between birth weight and subsequent litter size has a genetic basis; indeed, the response remains essentially unaltered if we were to remove the random effects (i.e. dam and ram) from the model, providing additional evidence for a lack of any residual genetic basis for our observed effect of birth weight on fertility.

## 3. A critique of existing evidence for the early-life programming of fecundity

### (a) Sheep and cattle

A series of studies conducted in Aberdeen, Scotland, during the late 1990s assessed the effects of global maternal nutrient restriction (typically between 50 and 70% requirements) during pregnancy on aspects of male and female foetal hypothalamic–pituitary–gonadal physiology in sheep (Rae *et al*. 2001, 2002). These studies reported some modest effects (e.g. reduction in foetal ovarian mass and germ cells; Rae *et al*. 2001); differential effects in terms of foetal luteinizing hormone response to gonadotropin releasing hormone challenge, which was restricted to males (Rae *et al*. 2002), but little else. Follow-up studies in pre- and post-pubertal animals found no long-term effects on the hypothalamic–pituitary axis (Borwick *et al*. 2003) and no effect of sperm production and quality of young rams (Rae *et al*. 2002), although there was an apparent reduction in the ovulation rate in young adult female offspring. A more robust statistical analysis of the data would help confirm this latter observation. For example, it was not clear how the size of the litter, from which these animals were derived, was accommodated in the analyses; this could denote a genetic effect on follicle development and ovulation rate, both of which are well established (Sinclair & Webb 2005). Significantly perhaps, and unlike the data presented in the current article, there was no evidence of intrauterine growth restriction (IUGR) following nutrient restriction, so that perhaps the modest effects observed were owing to the timing and/or severity of nutrient restriction. In a separate sheep model of IUGR involving over-nourished adolescent ewes, more compelling reductions in the size of the ovarian follicular pool were reported (Da Silva *et al*. 2001, 2002, 2003). Although Sertoli cell number was unaltered in the latter of these two studies, these authors had earlier reported a reduction in both testosterone production and testicular volume in pubertal male offspring from over-nourished adolescent dams (Da Silva *et al*. 2001). However, neither the onset of puberty nor subsequent ovarian cyclicity was altered in female offspring in that study.

The only previous study to convincingly demonstrate an impaired adult fertility-related phenotype in sheep investigated the effects of nutrient restriction during the first year of life (Gunn 1983). In that study, both ovulation rate and litter size of adult ewes were reduced. However, although reproductive data in that study were adjusted for body condition score at mating, there was no information on body fat distribution in these animals and, as will be discussed later, this could have had a bearing on pregnancy outcomes. A reanalysis of the data of Gunn *et al*. (1995), who assessed the effects of supplementary feeding during pregnancy or lactation on offspring fecundity over three successive parities, confirmed the importance of early post-natal growth relative to *in utero* development in determining adult fecundity (Gardner *et al*. 2008).

In contrast to sheep, there have been very few studies in cattle examining the effect of a potentially ‘adverse’ early environment on subsequent reproductive function—an important area of research for the dairy industry. In the multiparous dairy cow, the period encompassing late embryonic and early foetal development coincides with a significant competitive demand of a pronounced lactation, leading some investigators to assess the effects of the early nutritional environment on subsequent reproductive success in dairy heifers (Pryce *et al*. 2002; Swali & Wathes 2006). In both of these studies, there was no effect of maternal nutritional status during pregnancy on the reproductive performance of daughters. Collectively, therefore, these robust observations in ruminants suggest that while the developing foetal ovary, like most developing organs, may be susceptible to variations in maternal (and by inference, foetal) nutrition (Rae *et al*. 2001; Lea *et al*. 2006), there is little evidence to suggest that this pre-natal compromise in the development of the reproductive axis in either the sheep or cow translates into any significant functional deficit in reproductive performance during subsequent adulthood.

### (b) Rodents

In contrast to ruminants, the outcome of environmental perturbations on the reproductive axis of the rodent is likely to be more striking as they are poly-ovular; here, ovulation rate may be reduced in order to moderate litter size when faced with a long-term reduction in nutrient supply. This has been demonstrated experimentally when colonies of rats were maintained on a protein-restricted (6.8 versus 10% casein) diet for 12 successive generations (Stewart *et al*. 1975). By the 10th generation, females were significantly smaller (by approx. 15%), and average litter size was reduced from 10.7 ± 0.3 to 8.6 ± 0.2 (mean ± s.e.). In addition, sexual maturity was significantly delayed in the undernourished colony; whereas controls would mate at between eight and nine weeks, no female from the undernourished colony was able to conceive prior to the 14–16th week. Clearly, in this unique cohort of long-term marginally protein-restricted rats there were significant effects on the reproductive axis, but fertility *per se* was evidently unaffected; that is, adult females were still able to become pregnant and have many offspring. Furthermore, litter size greatly improved following rehabilitation, indicating no ‘programmed’, long-lasting effect. Given that, in altricial mammals, the investment in reproductive effort (i.e. annual fecundity rate) is 10 times that of humans (Phelan & Rose 2005), then the study of Stewart *et al*. (1975) illustrates the relative ‘protection’ afforded to fecundity even in these rather extreme species.

Indeed, there is much evidence from rodent models studied in the F_1_ generation after nutritional compromise during development (either during gestation and/or lactation) that the progeny exhibit evidence of delayed reproductive maturity, such as delayed puberty and increased reproductive cycle length (Guzman *et al*. 2006). Overnutrition (of *n*−6 polyunsaturated fatty acids), by contrast, resulted in an earlier onset of puberty (Hilakivi-Clarke *et al*. 1997). Furthermore, in the rat, exposure to a low-protein diet during gestation alone was sufficient to reduce sperm count and influence their ability to impregnate female rats in the F_1_ male offspring (Zambrano *et al*. 2005). Since rodents are born in an altricial state and experience a significant period of development outside the womb, this vulnerable developmental period has also been exploited experimentally: reduced maternal protein at this time alone was found to impact upon the female reproductive system in terms of reduced primordial follicle number (Silva Faria *et al*. 2008). As outlined earlier, such an effect has been observed in sheep, but only when the maternal diet was restricted pre-natally (Rae *et al*. 2001, 2002).

### (c) Non-human primates

A retrospective, multi-generational (data were available over a 40-year period) analysis was conducted on the breeding success of rhesus monkey females that were born small-for-date (SFD), average-for-date (AFD) or large-for-date and convincingly demonstrated that reproductive outcomes appear to pass down the matrilineal line (Price & Coe 2000). That is, SFD females were more likely to subsequently give birth to SFD females themselves, thus potentially perpetuating a maternal ‘constraint on growth’; no such effect was observed in the female offspring of AFD mothers or in the male offspring of SFD mothers. Other aspects of reproductive function influenced by having a mother born SFD were a delay to first conception (6 ± 0.3 versus. 5 ± 0.2 years), and higher rates of pre-term birth and neonatal mortality (Price & Coe 2000): effects remarkably similar to that reported by Stewart *et al*. (1975) in the female rat, discussed earlier.

## 4. Human fertility

In contrast to the animal studies referred to earlier, there are few studies of note in humans. The Dutch Winter Famine of 1944/45, however, presents a unique database in which to investigate effects on lifetime fecundity (Painter *et al*. 2005). During World War II, areas of the Netherlands were specifically isolated by the Nazis and food supply severely restricted for some four to five months. At the time of famine, some women were pregnant and the resultant offspring are now in their late middle age. Of the female babies that were born, many have now had their own families and thus investigation of potential effects on their reproductive health and fertility has been possible. To date, it would appear that markers of fertility such as age at first pregnancy, completed family size and inter-pregnancy interval from this cohort are not different from the non-exposed cohort of Dutch women (Lumey & Stein 1997; Lumey 1998). Indeed, a recent analysis of this cohort has even suggested an increased fertility (measured as average number of children for each famine-exposed woman), although the effect size reported (an increase from 1.7 to 2.0 (±1) children) was very small (Painter *et al*. 2008). Similarly, it has been shown that low birth weight (such as may occur following nutritional deficiency) does not advance the onset of reproductive senescence or menopause in women (Cresswell *et al*. 1997) but may reduce the age at which menarche occurs when coupled with greater post-natal weight gain (Sloboda *et al*. 2007). In addition, a recent study in women suggested that fecundity, represented by reproductive hormone levels and ovulation rate, may be inversely sensitive to ‘harsh’ environmental influences acting early in life (Nunez-de la Mora *et al*. 2007), supporting a developmental programming effect. However, the study could not delineate specific aspects of the harsh environment, such as nutritional factors or rates of infection, and whether actual lifetime fertility was reduced.

A common feature of *in utero* growth restriction, whether observed in mice (Ozanne & Hales 2004), rats (Widdowson & McCance 1975; Pitts 1986) or humans (Cameron *et al*. 2005), is the characteristic period of compensatory growth that normally follows during early childhood. This phenomenon has been proposed to underlie many of the adverse effects on cardiovascular function and metabolism observed during adulthood (Singhal & Lucas 2004; Cameron *et al*. 2005). The impact of catch-up growth on fertility in humans, however, is largely unknown. It certainly complicates interpretation of the effects of nutrient restriction during pregnancy *per se* on physiological function in the offspring. A key feature of compensatory growth is that rapid weight gain during childhood appears to partition energy disproportionately to abdominal adipose tissue (Wells 2007), which is thought to be causally linked to metabolic syndrome (Despres & Lemieux 2006). This is significant because it is quite probable that the increased prevalence of anovulation in adolescent girls born SGA compared with controls (40 versus 4%), and the reduced ovulation rate in ovulatory girls (1.4 versus 1.9) (Ibanez *et al*. 2002*b*), may be a consequence of deranged metabolism associated with central adiposity (Ibanez *et al*. 2002*a*) and not as a direct consequence of *in utero* nutrient restriction. SGA girls in the studies above never fully regained their target stature, but in a related study, visceral adiposity was greater and insulin resistance increased further in the SGA children who subsequently underwent catch-up growth (Ibanez *et al*. 2009). Nevertheless, the SGA girls reported by Ibanez *et al*. (2002*a*,*b*) were abdominally obese, hyperinsulinaemic and dyslipidaemic, all traits that were corrected following three months of metformin (an insulin-sensitizing drug) therapy (Ibanez *et al*. 2002*a*). Importantly, metformin treatment restored ovulatory activity in 70 per cent of subjects within 11 weeks of treatment, indicating that the reproductive pathology was linked to metabolism, was reversible and not permanently altered by impaired *in utero* development. In the study of Nunez-de la Mora *et al*. (2007) referred to earlier, it is possible that the migrant Bangladeshi women experienced a nutrition transition that led to secondary metabolic effects, as is common in men and women of Indian descent (Yajnik 2000, 2004), that influenced fecundity, rather than the harsh early environment *per se*.

**Figure 5.**
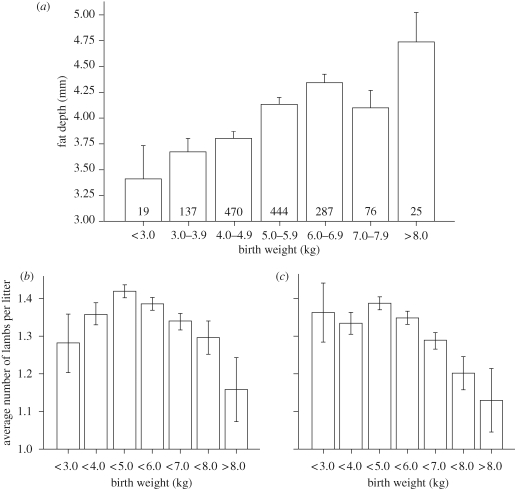
(*a*) Fat depth grouped according to birth weight category, and birth weight effects on fecundity (*F* = 7.10, *p* < 0.001 for linear trend), (*b*) unadjusted or (*c*) adjusted for maternal adiposity. Values in boxes represent the number of ewes in that category. The quadratic relationship between birth weight and fecundity was significant when unadjusted for maternal fat depth (*F* = 12.1, *p* < 0.001) and remained significant, but was weakened when adjusted for maternal fat depth (*F* = 9.8, *p* = 0.002).

In our cohort of pedigree sheep, it is a standard industry practice to ultrasound scan 20-week-old sheep for subcutaneous fat depth. As an indicator of overall adiposity, the measure correlated well with computerized tomography-determined adiposity (*r* = 0.72; *p* < 0.0001, *n* = 228 observations). Therefore, we examined the influence of female-offspring birth weight on her subsequent adiposity and lifetime fecundity. As birth weight increased so did adiposity at 20 weeks of age; for every 1 kg increase in birth weight, the subcutaneous fat depth increased by 0.27 ± 0.01 mm (3.67 (2.67–4.970), median interquartile range) (figure 5*a*). This observation in sheep is also apparent in humans; larger babies tend to become larger adolescents and overweight adults (Curhan *et al*. 1996; Stettler *et al*. 2000; Mamun *et al*. 2009). It is possible, therefore, that the reduced fecundity in our cohort of sheep reflected the fact that the mothers had greater adiposity. To address this issue, we fitted, as before, a model with fecundity as the response variate, birth weight category as the explanatory factor but subcutaneous fat depth as a covariate. The response curve remained inversely linear at birth weights above 5 kg but, at the lower end of the range (i.e. the SGA lambs), the response was lost, indicating that the decrease in fecundity with low birth weight is linked to the subsequent adiposity of the adult mother and is not primarily influenced by environmental factors contributing to low birth weight *per se*. This is a contention supported by the work of Ibanez (Ibanez *et al*. 2002*a*, 2009). Thus, in contrast to the larger lambs at birth, the reduced fecundity of SGA offspring may be associated with factors linked to our measured covariate, subcutaneous fat depth, such as altered insulin resistance and distribution of body fat (Yajnik 2004; Despres & Lemieux 2006; Botton *et al*. 2008). Indeed, it has been shown recently that carrying multiple copies of the fat-mass-associated gene variant FTO confers a greater risk of succumbing to polycystic ovarian syndrome, which reduces fertility (Barber *et al*. 2008). Nevertheless, our analyses are not fully bivariate and therefore cannot conclusively rule out the possibility of a residual genetic effect accounting for increased birth weight and reduced fecundity in our cohort of pedigree Suffolk sheep.

## 5. Conclusions

Taken together, the available evidence suggests that maternal nutrient restriction in mammals may have variable effects on gonadal development in the female foetus and some delayed programmed effects on the basic function of the female offspring's hypothalamic–pituitary–gonadal axis, but reproduction (i.e. lifetime fecundity) appears largely to be a protected function. This is emphasized by the failure to observe significant decrements in fecundity in two species with contrasting reproductive strategies: i.e. rats marginally protein-deprived over many generations (Stewart *et al*. 1975), and in female SGA non-human primates where their reproductive history was followed for 40 years (Price & Coe 2000). However, in this article and using a dataset from pedigree sheep, we show for the first time, to our knowledge, a curvilinear relationship between fecundity and early-life events, with apparent decreasing litter size at either end of the birth weight range. While we cannot fully dismiss residual genetic contributions, the curvilinear relationship in itself may be the reason for why many studies looking at extremes report no effect of birth weight on litter size or lifelong reproductive performance. However, in adjusting our data for body fatness during adolescence, the reduction in litter size associated with reduced birth weight was lost, indicating that the effects of small size at birth on reproductive performance may be secondary to reduced body fatness during adolescence. By contrast, the negative relationship between large size at birth and reduced litter size appears to be largely independent of adolescent body composition. Nevertheless, the available evidence would indicate that there is at best a minimal effect of maternal nutritional status during pregnancy on the lifelong reproductive performance of offspring.
